# Postoperative S100B protein levels as a measure for predicting neurological deficit in children undergoing cardiac surgery

**DOI:** 10.3389/fcvm.2026.1793108

**Published:** 2026-06-08

**Authors:** Evyatar Hubara, Lital kalich Philosoph, Simon Lassman, Yelena Skourikhin, Rina Hemi, Omer Bar-Yosef

**Affiliations:** 1 Pediatric Cardiac Intensive Care Unit (PCICU), Edmond and Lily Safra Children's Hospital, Sheb, Ramat Gan, Israel; 2 Weinberg Child Development Center, Edmond and Lily Safra Children's Hospital, Sheba Medical Center, Ramat Gan, Israel; 3 Pediatric Neurology, Sheba Medical Center, The Edmond and Lily Safra Children's Hospital, Ramat Gan, Israel; 4 Endocrine Laboratory Institute of Endocrinology, Sheba Medical Center, Ramat Gan, Israel; 5 Sackler Faculty of Medicine, Tel Aviv University, Tel Aviv, Israel

**Keywords:** biomarker, brain, congenital-heart-defect, injury, neurology, S100B

## Abstract

Brain injury following congenital heart surgery is associated with long-term neurodevelopmental deficits. Early identification of such injury may guide timely neuroprotective interventions. The calcium-binding protein S100B has been established in retrospective studies to be associated with poor neurological outcomes after surgery. These findings suggest that S100B may serve as a biomarker of central nervous system injury. Our study prospectively investigates the predictive value of S100B as a marker for new neurological deficits (NND) in infants undergoing cardiac surgery. In a prospective cohort of 130 infants undergoing congenital heart surgery, S100B was sampled before (*t* = 0, S100B1) and six hours after surgery (*t* = 6, S100B2). Neurological function was assessed using the Pediatric Stroke Outcome Measure (PSOM) before surgery and at discharge. A PSOM increase of ≥ 1 point was defined as a positive NND result. S100B levels were normalized by age (z-scores), and ROC analysis was used to evaluate predictive performance and determine the optimal diagnostic cut-off value. The S100B2 z-score showed the highest predictive value for NND with 93% sensitivity (28/30), 85% specificity (85/100), a negative predictive value of 0.98 (85/87), a positive predictive value of 0.65 (28/43), and a likelihood odds ratio of 79.3. A threshold of two standard deviations provided the best diagnostic accuracy. In multivariate analysis, the S100B2 z-score remained the strongest independent NND predictor. Age-normalized S100B levels measured 6 h post-surgery can serve as a marker to identify or exclude early brain injury leading to NND, supporting their use in post-surgical clinical decision-making.

## 1 Introduction

Congenital heart defects (CHD), with a prevalence of 6–9 children for 1,000 live birth, represent the most common congenital disorder worldwide (1). Approximately one-third of affected children require surgical or other invasive interventions during early infancy (2). These procedures carry a significant risk of brain injury, which is strongly associated with subsequent neurodevelopmental deficits. While advances in surgical techniques have improved survival rates, cardiac surgery is considered a major risk factor for neurological complications. Infants with CHD are more prone to neurocognitive impairments after surgery compared to infants, toddlers and children without CHD (3–5). This increased risk is largely attributed to perioperative factors such as the timing of surgery, need for resuscitation, seizures and brain injury and prolong hospitalization period (6–9).

In the last decades, several studies have explored a series of serum biomarkers that may serve as early indicators to predict the extent of brain damage after cardiac surgery to minimize its effect (10–12). However, the practical applicability of these markers in guiding patient management remains under investigation (13). Among them, the S100B protein has emerged as one of the most promising candidates.

S100B is one of the damage-associated molecular pattern molecules, primarily expressed in the central nervous system (CNS) by astrocytes, maturing oligodendrocytes, and certain neuronal populations (14, 15), and it may also play a role in myocyte apoptosis. S100B serum concentration is age-dependent and usually decreases with increasing age (16, 17). At high concentrations, it becomes neurotoxic and contributes to the pathophysiology of neurodegenerative disorders (18, 19). Moreover, the relation between high S100B levels and poor neurologic outcomes was demonstrated in several pediatric conditions (20–22). As noted, S100B has the potential to serve as an early biomarker for brain injury associated with poor neurological outcomes in children undergoing cardiac surgery, especially in the immediate post-operative period (23). Additionally, several studies reported that infants who experience early complications, such as death or brain damage, during the perioperative period show significantly higher S100B levels compared to infants without perioperative complications (24–27).

Despite its potential, the role of S100B as a prognostic marker for brain damage during the perioperative period remains controversial (28), as the protein is also expressed in extra-CNS cell types, such as adipocytes, chondrocytes, and melanocytes (29). This is particularly relevant to cardiac surgery, where damage to the mediastinal fat pad may lead to the release of S100B into systemic circulation (18–30). However, studies in CHD infants suggest that extracranial sources do not significantly affect serum levels of the S100B protein (31, 32). As most studies on S100B are case-control in design, the aim of the present study was to help clarify its utility as a prognostic factor for neurological deficit.

The aim of this study is to evaluate the efficacy of S100B as a marker for brain injury in a cohort of children after cardiac surgery. The first objective is to determine the optimal S100B cut-off for diagnosing post-surgical brain injury associated with neurological deficits. The second step is to identify additional factors that may enhance the prediction of brain injury.

## 2 Materials and methods

### 2.1 Study design and population

This study is a historic prospective cohort conducted in the Edmond J. Safra International Congenital Heart Center, located within the Edmond and Lily Safra Children's Hospital, at Sheba Medical Center, Israel, between the years 2015–2019. All female and male patients with congenital heart defects who were admitted for cardiac surgery between the ages of one day and eleven years were offered participation in the study. Patient data, including demographics, surgical details, hospitalization course, blood tests, and other clinical information, were gathered from electronic medical records.

### 2.2 S100B measurement

All patients included in the study were sampled for S100B on the day of the surgery, once before surgery (*t* = 0, S100B1) and again six hours after surgery (t = 6, S100B2). Blood samples were centrifuged at 1500 G for 10 min, and the extracted serum was stored at −80 °C until analysis. S100B levels were measured by a commercial immunoluminometric assay in a Liaison®Analyzer (Liaison®, DiaSorin S.p.A., Saluggia, Italy). The assay's calibration curve was linear up to 30 μg L^−1^, and the lower limit of detection is 0.02 μg L^−1^.

As S100B is known to vary with age (16, 17), values were normalized to z-scores to account for age-related differences (16). After recording absolute levels of S100B, z-scores were calculated, using age-specific mean and standard deviation for age described by Cohen (6). The z-score was defined as:

$$\frac{S100B-\bar{x}\;(S100B\;\mathrm{for}\;\mathrm{age})}{\mathrm{Standard}\;\mathrm{Deviation}\;\mathrm{of}\;S100B\;\mathrm{for}\;\mathrm{age}}$$

This represents the number of standard deviations an individual's S100B level deviates from the expected mean for their age.

### 2.3 Pediatric stroke outcome measure (PSOM) scale

All patients underwent neurological assessments before and after surgery. Pediatric residents, trained and guided by a pediatric neurologist (33), evaluated the neurological status of the patients by using the Pediatric Stroke Outcome Measure (PSOM), which is adapted for age. The PSOM scale was chosen as most of the brain injuries in children after cardiac surgery are ischemic in nature, resembling stroke (33). The PSOM scale gives a baseline neurological deficit and function profile before surgery, allowing comparison with post-surgical outcomes. A new neurological deficit (NND) is defined as an increase of at least one point on the PSOM score at discharge compared to the baseline.

PSOM contains 115 test items and is applicable across a wide age range, from newborns to adults. It assesses neurological function across 5 subscales: right sensorimotor, left sensorimotor, language production, language comprehension, and cognitive/behavioral. Each subscale is scored as follows: 0 (no deficit), 0.5 (mild deficit with normal function), 1 (moderate deficit with decreased function), or 2 (severe deficit with missing function). The subscale scores are summed to yield a total score ranging from 0 (no deficit) to 10 (maximum deficit) (33). This structured evaluation, combined with professional neurological assessment, provides a reliable proxy for detecting brain injury. A one-point increase at discharge is considered indicative of NND.

### 2.4 Clinical definitions and risk classification

A complex heart defect is defined as including any of the following diagnoses: Tetralogy of Fallot, transposition of great arteries, hypoplastic left heart syndrome, coarctation of the aorta, aortic stenosisThese diagnoses represent approximately 80% of the cases in the cohort. The rest were either or atrial septal defect of ventricular septal defect.

Postoperative neurological events are defined as either abnormal neurological findings on clinical examination or abnormal brain imaging findings on ultrasound, CT, or MRI, including findings such as brain atrophy, hemorrhage, or stroke. Such events were reported in approximately 20% of the cases in the cohort.

Surgical eventrefers to potentially adverse intraoperative occurrences, such as cardiopulmonary resuscitation, failure to wean from cardiopulmonary bypass, arrhythmias, or major bleeding.

The Risk Adjustment for Congenital Heart Surgery (RACHS-1) score, which estimates the risk of in-hospital mortality following congenital heart surgery was assessed for all infants in the study (34).

### 2.5 Statistical analysis

Continuous variables were evaluated for normal distribution using histograms and Q-Q plots, and are presented as median and interquartile range. Categorical variables are reported as frequencies and percentages. Comparisons of continuous variables were performed using the Mann–Whitney test and the Kruskal–Wallis test. Categorical variables were compared using the chi-square test or Fisher's exact test. Changes in S100B concentration over time were analyzed using the Friedman test, followed by the Wilcoxon test. Correlations between continuous variables were evaluated using Spearman's correlation coefficient. The discriminatory ability of each S100B measurement to identify n eurological deficits was evaluated using receiver operating characteristic (ROC) curves and the corresponding area under the curve (AUC). All statistical analysis was performed using SPSS (SPSS (Statistical Package for the Social Sciences, IBM, Armonk, NY, USA).

## 3 Results

### 3.1 Epidemiological and hospitalization data

 Table 1 lists the epidemiological data, including the broad demographic characteristics, course of surgery, and post-surgical events. The study included 130 infants, with an approximately equal distribution of males and females. A total of 104 infants (80%) had a complex congenital heart defect, and 87 infants (67%) had a cyanotic heart defect. Cardiopulmonary bypass was used in 111 cases (85%). According to the RACHS-1 classification, 81 infants (63%) fell into categories 3–4, indicating an intermediate risk of mortality. A small proportion of patients (7%) required an Extracorporeal Membrane Oxygenation Machine (ECMO). The mean duration of hospitalization was 25.3 days, and the average Pediatric Cerebral Performance Category (PCPC) score increased from 1.1 ± 0.4 at admission to 1.5 ± 1.1 at discharge. NND was diagnosed in 30 children (23%), determined by the change in PSOM value.

**Table 1:** Epidemiological and hospitalization data.

Category	Variable	Value
Demographics	Female, N (%)	62 (48%)
	Cyanotic heart defect, N (%)	87 (67%)
	Complex heart defect, N (%)	104 (80%)
RACHS-1 Score	Score 1	10 (8%)
	Score 2	20 (16%)
	Score 3	46 (36%)
	Score 4	35 (27%)
	Score 5	0 (0%)
	Score 6	18 (14%)
Surgical History	Previous surgery, N (%)	20 (15%)
	Age at surgery, months (mean ± SD)	16.2 ± 29
	Weight, kg (mean ± SD)	7.3 ± 8.4
	Surgery duration, hours (mean ± SD)	3.2 ± 2.1
	Cardiopulmonary bypass, N (%)	111 (85%)
	Aortic clamp, N (%)	77 (59%)
	Intraoperative event, N (%)	27 (21%)
	ECMO, N (%)	9 (7%)
	Postoperative anesthesia, hours (mean ± SD)	14.7 ± 39.8
Post-Surgical Outcomes	Neurological event, N (%)	24 (18.5%)
	Hospitalization, days (mean ± SD)	25.3 ± 25.4
	Time to oral nutrition, days (mean ± SD)	9.2 ± 12.2
	Time to regain pre-surgery GCS^a^, days (mean ± SD)	7.4 ± 3.2
	Admission PCPC (mean ± SD)	1.1 ± 0.4
	Discharge PCPC (mean ± SD)	1.5 ± 1.1
	PSOM change ≥1, N (%)	30 (23%)

a Glasgow Coma Scale.

### 3.2 ROC analysis for the best S100B predictor of NND

**Figure 1:**
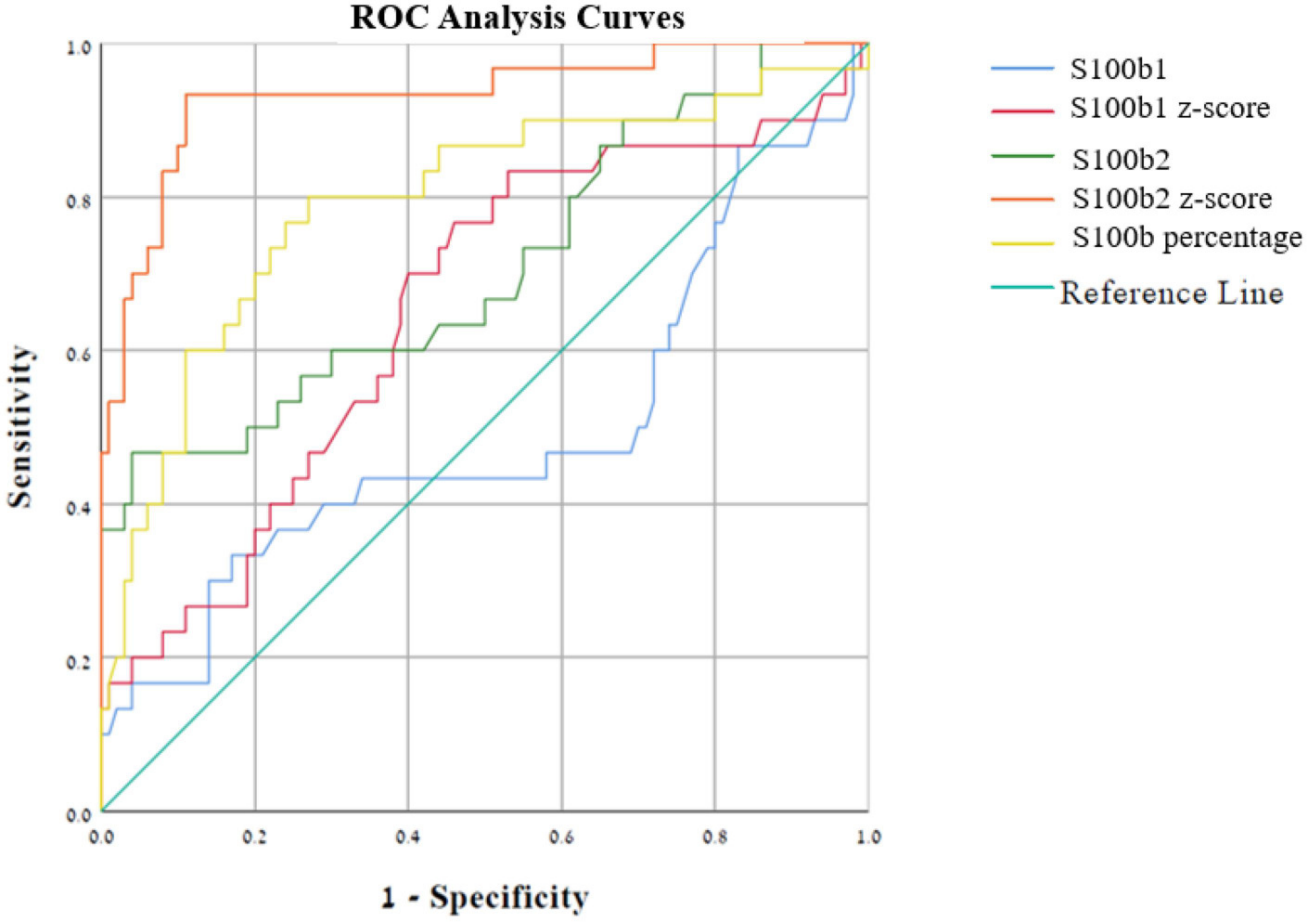
ROC curves comparing the predictive performance of S100B biomarkers for NND.

ROC analysis curves were generated to assess the ability of absolute and normalized S100B z-scores at baseline and 6 h post-surgery to predict NND. The AUC values are presented in Table 2 and illustrated in Figure 1. The normalized S100B2 z-score at 6 h post-surgery (S100B2-z-score) demonstrated the highest diagnostic accuracy, with a statistically significant AUC of 0.932 (*p* < 0.001), as defined in Section 2.5. This finding supports the diagnostic potential of the S100B2 z-scores for early identification of NND. Given the superior predictive power of the S100B2 z-score for NND, we further determined its optimal diagnostic cut-off value. Table 3 summarizes the sensitivity and specificity results of S100B2 z-score ranging from 1 to 3 standard deviations. A threshold of 2 standard deviations was identified as the most effective cut-off, providing the best balance between sensitivity and specificity.

**Table 2:** AUC for S100B before and after surgery.

Parameter	Area (SE)	*p*-value
S100B1	0.49 (0.07)	0.9
S100B1 z-score	0.65 (0.06)	0.01
S100B2	0.71 (0.06)	0.001
S100B2 z-score	0.93 (0.03)	0.0001
S100B percentage	0.79 (0.05)	0.0001

**Table 3:** Sensitivity and specificity at different S100B2 z-scores.

Standard Score	1	1.5	2	2.5	3
Sensitivity	0.93	0.93	0.93	0.87	0.73
Specificity	0.68	0.80	0.85	0.90	0.94

Table 4 presents detailed statistical results for the S100B2 z-score using a cut-off of 2 standard deviations. At this threshold, 28 out of 30 NND cases were correctly identified, while 85 out of 100 children without brain injury were accurately classified as negative. This resulted in a negative predictive value of 0.98 and a positive predictive value of 0.65. The analysis indicates that the odds of successfully identifying an NND using the S100B2 z-score at this cut-off are more than 79 times greater than without it.

**Table 4:** Statistical outputs for S100B2 z-score at 2 standard deviations.

Measure	Value (CI 95%)	Calculation
Sensitivity	0.93 (0.87–0.99)	28/30
Specificity	0.85 (0.77–0.91)	85/100
False Negative Rate	0.07 (0.01–0.22)	2/30
False Positive Rate	0.‏15 (0.09–0.24)	15/100
Positive Predictive Value	0.65 (0.49–0.79)	28/43
Negative Predictive Value	0.98 (0.92–0.99)	85/87
Likelihood Odds +	6.22 (3.86–10.01)	0.93/0.15
Likelihood Odds -	0.08 (0.03–0.13)	0.07/0.85
Likelihood Odds Ratio	79.33 (17.08–368.60)	6.22/0.08

CI, Confidence Interval.

### 3.3 Clinical parameters associated with neurological outcome

A secondary aim of the study was to assess additional clinical parameters associated with poor neurological outcomes. These included the epidemiological, surgical, and post-surgical characteristics presented in Section 3.1. Logistic regression was performed for each parameter to assess its association with the presence of NND. Only statistically significant results (*p* < 0.05) are presented in Table 5. A multiple logistic regression model was then constructed, incorporating age at surgery, duration of surgery, and the S100B2 z-score. Anesthesia and bypass duration are correlated with surgery duration. Among the parameters tested, only the S100B2 z-score remained significantly associated with NND, as seen in Table 6. Each one-unit increase in the S100b z-score was associated with a 1.23-fold increase in the odds ratio (OR) of NND.

**Table 5:** Clinical parameters associated with NND.

Parameter (mean ± SD)	No NND (*n* = 100)	NND (*n* = 30)	*p*-value
Age at surgery, months	12.6 ± 26.1	28.4 ± 35.5	0.005
Surgery Duration, hours	2.9 ± 2.0	4.0 ± 2.2	0.001
Anesthesia, hours	13.8 ± 41.1	17.4 ± 35.5	0.044
Ventilation, hours	93.4 ± 124.7	185.1 ± 212.0	0.049
Bypass duration, minutes	64.1 ± 47.6	98.8 ± 67.8	0.004

**Table 6:** Multiple logistic regression model.

Parameter	OR (CI 95%)	*p*-value
S100B2 z-score	1.23 (1.06–1.44)	0.006
Age at surgery: Month	1.02 (0.99–1.03)	0.103
Surgery: Hours	1.12 (0.91-.137)	0.286

## 4 Discussion

Previous studies have shown that S100B increases following cardiac surgery in children with NND or brain injury (23, 31, 35–45). This increase has been observed as early as 6 h post-operatively, implying the occurrence of perioperative brain insult (23). However, those studies were retrospective case-control studies, which did not allow evaluation of the predictive value of S100B. The present prospective cohort study aimed to assess the predictive capacity of S100B by initially deriving its optimal diagnostic cut-off and then evaluating its prognostic performance, both independently and in combination with other clinical and epidemiological parameters.

The proportion of NND in our study was 23% (30/130), consistent with previously published studies. Previous studies have published similar portion of peri-operative brain injury based on pre- and post-operative brain MRI scans (46–48). Even the most recent study from 2025 (49), demonstrates that from 192 children undergoing heart surgery, 23% had microhemorrhages in the brain pre-surgery and 85% post-surgery. These findings were associated with worse neurodevelopmental outcome at 18 months. A long-term follow-up study, Mussatto et al. (3) found that 35/99 children (35%) with CHD had significant developmental delay more than two years after surgery. Brosig et al. (4, 5) assessed development outcomes in pre-school children with CHD and found that, among children without a genetic disorder, up to 33% exhibited delays in at least two developmental domains. This proportion increased to 65% among children with an underlying genetic disorder. The similarity in NND prevalence between our study and previously published findings is highly important, as it strengthens the ability to generalize its results.

Using ROC analysis, we demonstrated that the age-normalized S100B level measured 6 h after surgery has strong predictive value for NND. This finding is consistent with previous case study (23). Moreover, a detailed comparison of diagnostic thresholds indicated that a cut-off of 2 standard deviations provides the best balance of sensitivity and specificity for predicting NND. The neurological status of children following cardiac surgery remains a major concern in the pediatric intensive care unit setting (PICU). Early diagnosis of potential neurological damage may raise clinical suspicion for complications such as brain hemorrhage, seizures, or delirium, prompting further investigation such as brain imaging or EEG and the initiation of protective treatment. Conversely, a normal S100B value may reduce the likelihood of brain injury, thereby helping to avoid unnecessary diagnostic procedures, such as post-operative brain MRI. The 2 standard deviation threshold of S100B measured 6 h after surgery appears to be a valuable tool for early diagnosis or exclusion of new brain injury leading to NND. Using this threshold, 93% of NND cases were correctly identified. Among the 48 children who crossed the threshold, 65% were correctly predicted to have NND, while 35% may have been over-evaluated or treated for brain injury without clear justification. This information limits the use of S100B as the sole marker of brain injury after surgery. However, in cases of high suspicion for brain injury due to of surgical complications (i.e., prolong CPB, air emboli) high S100B could support clinical decisions, as early imaging or neuroprotective treatment. As for the exclusion of brain injury, the negative predictive value of the S100B2 z-score was 98%. Overall, S100B may serve as a supportive tool for clinical decision-making in the PICU during the critical hours after surgery. These findings are in keep with studies on traumatic brain injury. These studies show that S100B below a very low cut off (0.1 µg/L) to role out brain hemorrhage and to reduce unnecessary brain CT (22). Given the high incidence of brain injury in this patient population, S100B represents a highly valuable biomarker for both raising suspicion and confidently ruling out new brain injury.

A multiple logistic regression model was constructed to test whether including clinical and epidemiological parameters could improve the prediction of NND. However, the S100B2 z-score emerged as the strongest independent predictor. Variables such as age at surgery and duration of surgery did not significantly enhance the model's predictive power.

Previous studies found that infants with different types of CHD exhibit varying levels of S100B (48). Thus, further categorization by specific heart defect subtypes is warranted, as their individual contributions to S100B levels remain insufficiently explored. For example, infants with cyanotic heart disease have been reported to have higher circulating S100B concentrations before surgery than infants with non-cyanotic heart disease (48), which may influence the interpretation of postoperative S100B dynamics. Differences in anatomical surgical cut may influence S100B level depending on the tissues involved (18, 30). To overcome these problems a serial S100B is suggested at the initiation of the surgery, at the end of the surgery and between 3 and 6 h after surgery. A significant, continuing increase of S100B after surgery could be biomarker for brain injury than a single sample.

## 5 Conclusion

Serum S100B levels show promise as a near real-time biomarker for early detection of neurological injury in children following cardiac surgery. A threshold of two standard deviations may help clinicians in both identifying early brain injury and confidently ruling out its occurrence. Access to additional diagnostic information during the immediate postoperative period is essential to raise clinical suspicion when warranted and to avoid unnecessary diagnostic procedures. To fully establish the clinical utility of S100B, its levels should be measured both preoperatively and immediately after surgery, with results made available to the clinician within hours. Only under these conditions can its true impact on clinical decision-making be adequately assessed.

## Data Availability

The original contributions presented in the study are included in the article/supplementary material, further inquiries can be directed to the corresponding authors.
